# Pro-Apoptotic Activity of French Polynesian *Padina pavonica* Extract on Human Osteosarcoma Cells

**DOI:** 10.3390/md16120504

**Published:** 2018-12-13

**Authors:** Giulia Bernardini, Mariagiulia Minetti, Giuseppe Polizzotto, Manuele Biazzo, Annalisa Santucci

**Affiliations:** 1 Dipartimento di Biotecnologie, Chimica e Farmacia (Dipartimento di Eccellenza 2018–2022), Università degli Studi di Siena, via Aldo Moro 2, 53100 Siena, Italy; bernardini@unisi.it (G.B.); minetti2@student.unisi.it (M.M.); 2 Institute of Cellular Pharmacology (ICP Concepts Ltd.), F24, Triq Valletta, Mosta Technopark, MST 3000 Mosta, Malta; giuseppe@icpconcepts.com (G.P.); manuele@icpconcepts.com (M.B.)

**Keywords:** *Padina pavonica*, osteosarcoma, apoptosis, algae, chemo-preventive agent, phytol, fucosterol, fatty acid

## Abstract

Recently, seaweeds and their extracts have attracted great interest in the pharmaceutical industry as a source of bioactive compounds. Studies have demonstrated the cytotoxic activity of macroalgae towards different types of cancer cell models, and their consumption has been suggested as a chemo-preventive agent against several cancers such as breast, cervix and colon cancers. Reports relevant to the chemical properties of brown algae *Padina* sp. are limited and those accompanied to a comprehensive evaluation of the biological activity on osteosarcoma (OS) are non existent. In this report, we explored the chemical composition of French Polynesian *Padina pavonica* extract (EPP) by spectrophotometric assays (total phenolic, flavonoid and tannin content, and antioxidant activity) and by gas chromatography-mass spectrometry (GC-MS) analysis, and provided EPP lipid and sterols profiles. Several compounds with relevant biological activity were also identified that suggest interesting pharmacological and health-protecting effects for EPP. Moreover, we demonstrated that EPP presents good anti-proliferative and pro-apoptotic activities against two OS cell lines, SaOS-2 and MNNG, with different cancer-related phenotypes. Finally, our data suggest that EPP might target different properties associated with cancer development and aggressiveness.

## 1. Introduction

Recently, seaweeds and their extracts have attracted great interest in the pharmaceutical industry as a source of bioactive compounds [1].

A number of studies have demonstrated the cytotoxic activity of macroalgae towards different types of cancer cell models and certain authors have suggested the consumption of algae as a chemo-preventive agent against several cancers. In particular, brown algae have demonstrated to be rich in unsaturated fatty acids, which block growth and systemic spread of human breast cancer, polysaccharides and terpenoids which are considered as promising bioactive molecules with anticancer activity [2,3]. *Padina pavonica* is representative of brown algae which can be found throughout the world from warm temperate to tropical locations, including: North Carolina to Florida in the United States, the Gulf of Mexico, throughout the Caribbean and tropical Atlantic and the Eastern Atlantic, Mediterranean and Adriatic Seas [4]. There are several species of algae belonging to the genus *padina*. The main chemical classes of compounds found in padina species are represented by: sterols, lipids, polysaccharides, carotenoids, polyphenols and fibers [5]. However, in addition to the name and the geographical spread, changes can be noted also in its biochemical composition.

Although Osteosarcoma (OS) is a rare disease comprising less than 1% of cancers diagnosed in the United States, it is the most common primary malignant bone tumor in adolescents and young adults. OS accounts for 8.9% of cancer-related deaths and carry an overall 5-year survival rate of 60–70% despite modern treatment protocols that combine chemotherapy and surgery [6,7]. Chemotherapy has been established as a critical component of OS therapy, but its adverse side effects associated with the drug resistance developed by tumors, lead to the urgent need for new and specific anticancer agents.

In this study, we aimed to demonstrate the antitumoral activity of the extract of *Padina pavonica* (EPP) on human OS cells in order to provide the molecular evidences supporting the development of EPP-based products usable as a potential chemo-preventive agent against OS.

## 2. Results

### 2.1. Chemical Composition and Antioxidant Capacity of Padina pavonica Extract

The extract of *Padina pavonica* (EPP) under investigation was produced by Soxhlet extraction using acetone as solvent, starting fronds of mature from algae collected in French Polynesia in June 2014.

EPP was first chemically characterized for its total phenolic, flavonoid and tannin content through spectrophotometric assays. The total phenolic, flavonoid and tannin contents of the seaweed were 27.0, 54.8, and 54.3 mg per g of extract, respectively, corresponding to 0.81, 1.64 and 1.63 mg per g of dry material, respectively. The antioxidant activity was evaluated by ferric reducing antioxidant power (FRAP) assay and resulted as 25.6 ± 0.2 µmol of Fe^2+^/100 mg of extract.

EPP was also examined for its lipid content by GC-MS. Hydrocarbons represented the 79.88% of the total extract, among which 68.83% corresponded to fatty acids (FAs), 0.19% corresponded to squalene and 10.86% to other hydrocarbon species (Table 1).

Sterols represented the 8.37% of the extract and included fucosterol and cholesterol in a percentage of 7.40% and 0.97%, respectively (Table 1). We also calculated the ratio between fucosterol and cholesterol (F:C) which corresponded to 7.6:1. Other noteworthy compounds identified were: α- and δ-tocopherol, which corresponded to 0.17 and 0.19% respectively, phytol (5.27%), neophytadiene, a terpene compound that amounted to 5.56%, 2,4-di-*tert*-butylphenol (0.18%) and dihydroactinidiolide (0.37%) (Table 1).

GC-MS analysis was also performed with a different sample preparation approach consisting in saponification and subsequent extraction by dispersive liquid-liquid microextraction (DLLME) of EPP, in order to analyse the most lipophilic compounds. This analysis mostly confirmed the presence of several already identified compounds (Table 2), such as: phytol (53.85%), fucosterol (17.57%), palmitic acid (12.00%), cholesterol (4.57%), 2,4-di-tert-butylphenol (2.99%), stearic acid (3.40%), oleic acid (0.59%), dihydroactinidiolide (0.62%), squalene (0.19%) and δ-tocopherol (0.27%), and n-nonadecanol-1 (0.20%).

EPPs FAs profile showed the presence of FAs with aliphatic chains ranging from 12 up to 22 carbon atoms (Table 3). Saturated FAs (SFAs) corresponded to 43.45% of total EPP (63.13% of total FAs). Among these, the most abundant FA was palmitic acid with a total percentage of 34.15%, followed by stearic (3.25%), pentadecanoic (1.95%), arachidic (0.74%), myristic (0.43%), lauric (0.47%) and behenic (0.04%). Monounsaturated FAs (MUFAs) were 23.67% of total EPP (34.40% of total FAs). The most abundant MUFA was palmitelaidic acid (16:1 *n*-7 E, 7.82%) followed by oleic acid (18:1 *n*-9, 7.79%) and palmitoleic acid (16:1 *n*-7 Z, 6.29%). Polyunsaturated FAs (PUFAs) corresponded to 1.70% of EPP (2.47% of total FAs). The main PUFAs found in EPP was arachidonic acid (20:4 *n*-6, 0.64%) followed by linoleic (18:2 *n*-6, 0.53) and eicosapentanoic acid (20:5 *n*-3 0.24%). We also calculated the ratio between *n*-6 and *n*-3 PUFAs which corresponded to 5.61.

### 2.2. EPP Effects on OS Cell Viability and Proliferation

**Figure 1:**
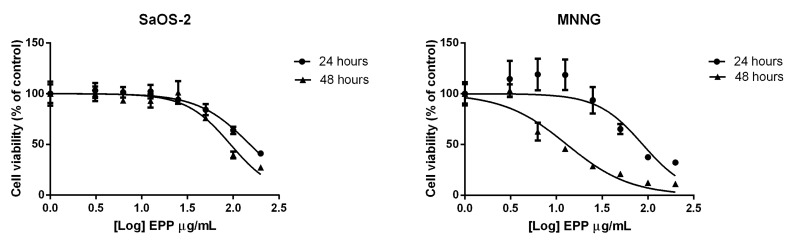
IC_50_ of EPP after 24 and 48 h treatment on SaOS-2 and MNNG. IC_50_ was calculated using Graphpad. Cell viability was expressed as percentage in respect to control and EPP concentrations (3.1, 6.25, 12.5, 25, 50, 100 and 200 μg/mL) were reported in a logarithmic scale.

EPP inhibited SaOS-2 cell viability in a dose and time dependent trend with an IC_50_ after 24 h treatment of 152.2 ± 7.7 µg/mL (Figure 1). These results were confirmed also after 48 h treatment.

On MNNG cells EPP showed a more pronounced effect on cell viability in respect of SaOS-2 cells, with a IC_50_ of 87.75 ± 18.57 µg/mL after 24 h treatment and a more remarkable effect was detected after 48 h treatment.

**Figure 2:**
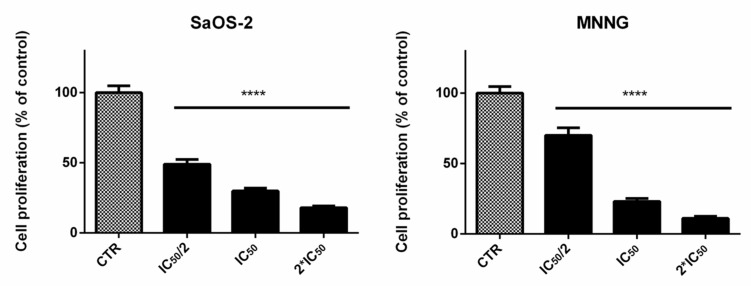
SaOS-2 and MNNG cell proliferation after 24 h treatment with EPP at IC_50_/2, IC_50_ and 2*IC_50_ in starved conditions determined by the Scepter 2.0 cell counter. Data are expressed as percentage in respect to control and presented as mean ± SD, **** *p* < 0.0001.

EPP induced a decrease of proliferation in SaOS-2 and MNNG cells in a dose-dependent manner (Figure 2). A reduction of cells proliferation of about 51%, 70% and 82% was observed for SaOS-2 when treated for 24 h with EPP at IC_50_/2, IC_50_ and 2*IC_50_ (as calculated by cell viability assay for SaOS-2) respectively. Analogously, in MNNG cells we observed a reduction in cells count of about 30%, 77% and 89% when treated for 24 h with EPP at IC_50_/2, IC_50_ and 2*IC_50_ (as calculated by cell viability assay for MNNG) respectively.

### 2.3. EPP Effects on OS Cell and Nuclear Morphology

**Figure 3:**
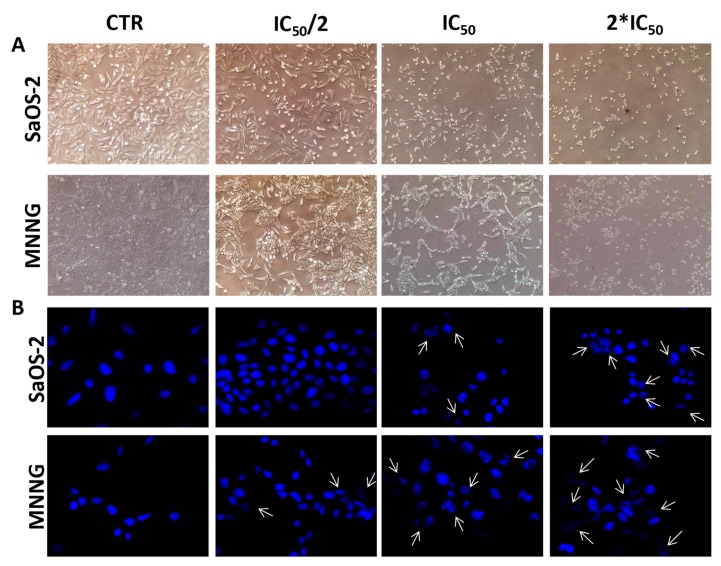
(**A**) Bright-field images of SaOS-2 and MNNG OS cells line after 24 h treatment with EPP at IC_50_/2, IC_50_ and 2*IC_50_ or DMSO 0.3% as negative control. Cells are shown at ×10 magnification. (**B**) Nuclear morphological changes and DNA damage assessment in SaOS-2 and MNNG cells OS cells line after 24 h treatment with EPP at IC_50_/2, IC_50_ and 2*IC_50_ using DMSO 0.3% as negative control. Arrows indicate nuclear fragmentation, which can be considered a biochemical hallmark of apoptosis. Cells are shown at ×63 magnification.

Bright-field images showed noticeable morphological changes in both OS cell lines, moving from control to the highest concentration of EPP (Figure 3A). After 24 h treatment with EPP at IC_50_ and 2*IC_50_, cells lost their original elongated shape and become rounding and blebbing. A reduction in cell number and dimension, as well as cytoplasm condensation were also observed in both SaOS-2 and MNNG cells, representing a clear sign of the activity of the treatment.

To evaluate whether EPP exhibited cytotoxicity through apoptosis in both OS cell lines, a DAPI staining analysis was performed to observe nuclear morphological changes (Figure 3B). Such analysis demonstrated that the exposure of OS cells to EPP induced apoptosis in a dose dependent manner; indeed, both SaOS-2 and MNNG cells showed loss of regular shape and well-defined boundaries. Moreover, at the highest concentration tested (2*IC_50_), EPP exhibited a more remarkable apoptotic effect against MNNG than SaOS-2 with greater nuclear fragmentation, chromatin condensation and nuclear blebbing.

These evidences were confirmed by the high percentage of late apoptotic MNNG cells rather than SaOS-2 cells (*par*. 2.4).

### 2.4. EPP Induces Apoptosis in OS Cells

**Figure 4:**
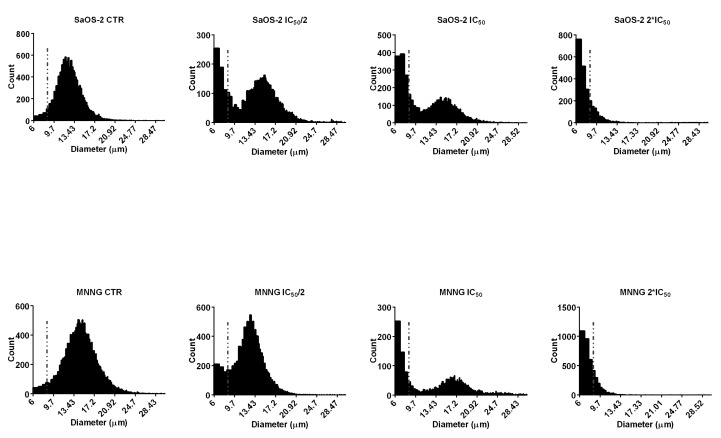
Diameter distributions of SaOS-2 and MNNG cells after 24 h treatment with EPP at IC_50_/2, IC_50_ and 2*IC_50_ as measured by Scepter 2.0 cell counter. The cells were classified into small (diameter: 6–9 µm) or large (diameter 9–24 µm) fractions depending on the gating range.

The exposure of SaOS-2 and MNNG cells to EPP resulted in dose-dependent increase of the percentage of small diameter cells (6–9 µm), compared to control (Figure 4). The gradual increase in the percentage of cells in the small particle fraction after treatment with different concentrations of EPP can be regarded as an index of late cell apoptosis.

**Figure 5:**
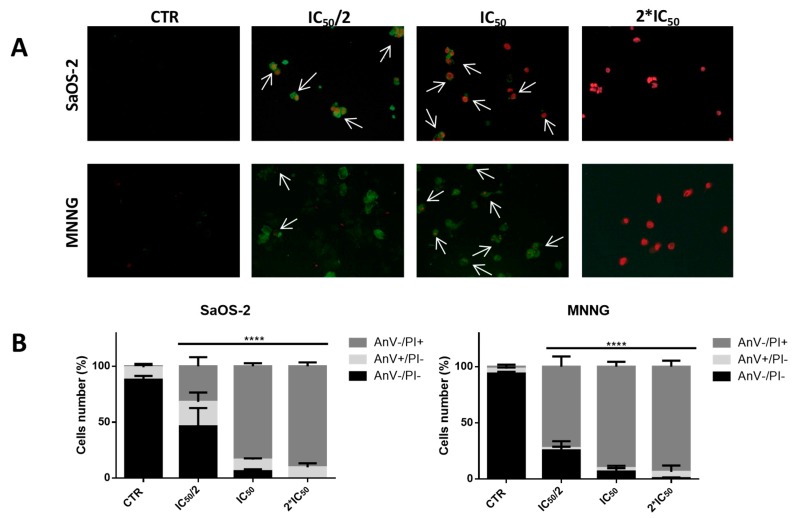
(**A**) Apoptosis assessment using Annexin V-FITC/PI staining and fluorescent microscopy of SaOS-2 and MNNG OS cells line treated with EPP/Acetone (IC_50_/2, IC_50_, 2*IC_50_) for 6 h. Viable cells did not show any kind of coloration (Anv−/PI−). Cells stained in green (AnV+/PI−) were considered as early apoptotic cells, while cells stained both in green and red (Anv+/PI−) were considered as late apoptotic cells. Cells are shown at ×40 magnification. Arrows indicate co-localization. (**B**) Histograms show the percentage of non-apoptotic(AnV−/PI−), early apoptotic (AnV+/PI−) and late apoptotic (AnV+/PI+) cells in respect to control. The quantitative assessment of apoptosis was obtained observing merged images. Results were obtained from three different experiments in triplicate, **** *p* < 0.0001.

To confirm the pro-apoptotic effect of EPP, we performed the Annexin V-FITC/propidium iodide (PI) assay on SaOS-2 and MNNG cells treated with EPP (at their relative IC_50_/2, IC_50_ and 2*IC_50_) for 6 h (Figure 5A,B and Table 4). SaOS-2 apoptotic cells were, roughly, completely absent in the untreated culture, as expected for cancer cells: 88.04 ± 3.33% of non-apoptotic (AnV−/PI−), 11.17 ± 2.94 of early apoptotic (AnV+/PI−) and 0.79 ± 0.47% of late apoptotic (AnV+/PI+). When cells were treated with progressively higher concentrations of EPP, the number of early apoptotic and late apoptotic cells increased in a dose-dependent manner (IC_50_/2: 21.80 ± 8.31% of AnV+/PI− and 31.83 ± 8.20% of AnV+/PI+; IC_50_: 10.46 ± 1.07% of AnV+/PI and 83.43 ± 2.76% AnV+/PI+; 2*IC_50_: 9.60 ± 3.53% of AnV+/PI and 90.40 ± 3.53% of AnV+/PI+). MNNG cells (Figure 5A,B) had a quite similar trend, finding a very low percentage of apoptotic cells (6.08 ± 2.84%) in the untreated culture and an increasing percentage of early and late apoptotic cells with progressively higher concentrations of EPP (IC_50_/2: 2.05 ± 1.26% of AnV+/PI− and 72.46 ± 9.12% of AnV+/PI+; IC_50_: 2.99 ± 2.12% of AnV+/PI and 90.34 ± 4.27 AnV+/PI+; 2*IC_50_: 5.63 ± 5.63 of AnV+/PI and 93.67 ± 5.32% of AnV+/PI+). On MNNG cells, EPP demonstrated a greater pro-apoptotic effect. In fact, in MNNG cells treated with IC_50_/2, we detected a greater percentage of late apoptotic cells in respect of SaOS-2 challenged with the same EPP concentration (AnV+/PI+: 72.46 ± 9.12% and 31.83 ± 8.20%, respectively).

**Figure 6:**
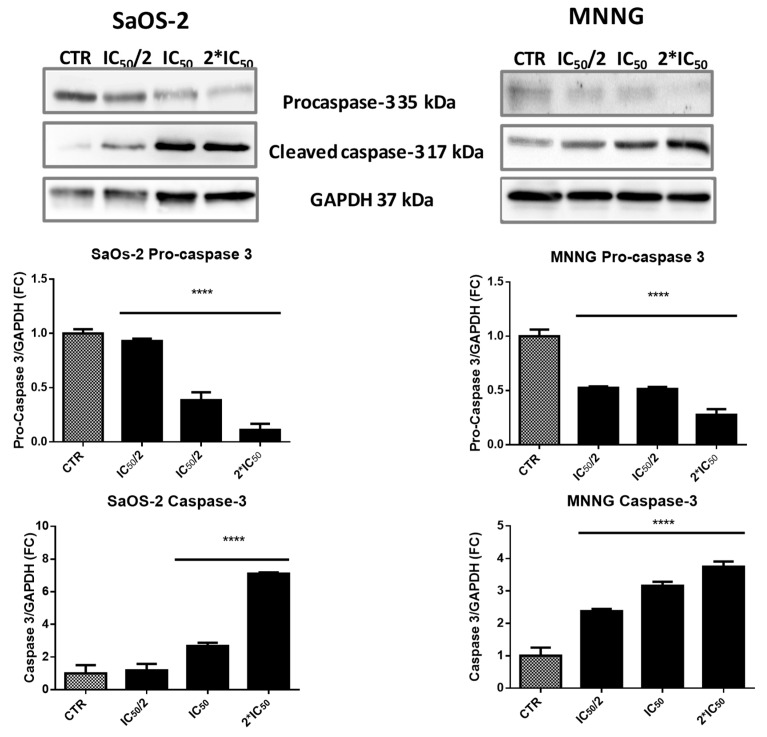
EPP treated SaOS-2 and MNNG OS cells line western blotting images of procaspase-3, cleaved caspase-3 and GAPDH. Data are expressed as fold-change in respect to control and presented as mean ± SD. Results were obtained from three different experiments in triplicate, **** *p* < 0.0001.

To study if caspases activation was involved in EPP-induced apoptosis, we finally evaluated the activation of caspase-3 by western blot analysis. The immunoreactive band of cleaved caspase-3 increased in a dose-dependent manner after 6 h treatment with EPP (Figure 6). In SaOS-2 cell lines we detected a 2.6 and 7.1-fold increase in the activation of caspase-3 when treated with EPP at IC_50_ and 2*IC_50_ respectively. On the contrary in MNNG cells, we observed a dose-dependent increase in the activation of caspase-3. The immunoreactive band was significantly higher than SaOS-2 already after treatment with the lowest concentration of EPP (2.4-fold at IC_50_/2) and increased over the other two concentrations (3.2-fold at IC_50_ and 3.7-fold at 2*IC_50_).

Lower panel: Graph reports the decrease of procaspase-3 and the increase of cleaved caspase-3 in a dose dependent manner after 6 h of treatment with IC_50_/2, IC_50_ and 2*IC_50_ EPP. Values are calculated as a ratio of band volume of procaspase-3 or caspase-3 over band volume of GAPDH.

## 3. Discussion

Recently, natural bioactive compounds derived from marine organisms, especially those obtained from seaweeds, have received greater attention. Their high level of biodiversity makes them a considerable reservoir for active compounds as they are able to produce a great variety of secondary metabolites characterized by a wide range of biological activities. Many previous studies demonstrated the remarkable benefits of seaweeds on human health and protection against chronic disease [8] due to their content in proteins, lipids and fatty acids, polysaccharides and antioxidant compounds. It has been demonstrated that fatty acids extracted from marine algae block growth and spread of human breast cancer [9]. In addition, polysaccharides and terpenoids from brown algae have demonstrated to be promising bioactive molecules with anticancer activity [3].

In this work, for the first time the acetonic extract of *Padina pavonica* (EPP), a brown seaweed collected from French Polynesia, was chemically characterized and demonstrated to have a strong pro-apoptotic effect on human OS cells.

Reports relevant to the chemical properties of brown algae *Padina* sp. are limited and those providing a detailed description of the chemical profile accompanied to a comprehensive evaluation of the biological activity for *Padina pavonica* from French Polynesia are nonexistent.

We first characterized EPP for its total phenolic, flavonoid and tannin content, providing the first chemical report on French Polynesian *Padina pavonica*.

In our extract, total phenolic compounds amounted to 27 mg per g of extracts (0.81 mg per g of dry material). Considering the acetone as the solvent used for the extraction, our is a remarkable quantity if compared to total phenolic compounds found by Khaled et al. in Lebanese *Padina pavonica* (10.76 mg per g of the methanolic extract) [10] and by CAF et al. in Turkish Mediterranean *Padina pavonica* (0.96 mg per g of the aqueous extract and 1.76 mg per g of the methanolic extract) [11]. Pinteus et al. in *Padina pavonica* from Portugal, found values of 44.61 and 10.48 mg per g of extract when using methanol and dichloromethane respectively as extraction solvents [12]. In their work Hlila et al. determined the total phenolic content of aqueous and acetonic extract of Tunisian *Padina pavonica*, with values corresponding to 57.34 and 90.61 mg per g of extract, respectively [13].

Phenolic compounds, one of the most important class of natural compounds, are commonly found in brown algae where they exert a protective effect toward adverse environmental conditions. They have been reported to possess several biological activities including anti-oxidant, anti-bacterial and anti-allergic and anti-diabetes, and to be involved in the protection against several human diseases such as cancer, coronary heart disease, inflammatory and neurodegenerative diseases and aging [14,15].

With regard to flavonoid content, *Padina pavonica* from French Polynesia appeared to be rich in flavonoids, showing a value of 54.8 mg per g of extract. This is a relevant data if compared to previous report for Australian *Padina* sp., which reported a total flavonoid content of 20.74 mg per g of ethanol extract [16]. Flavonoids are an important class of phenolic compounds, that contribute to the antioxidant activity of algae extracts.

About tannin content, their concentrations vary greatly among different species of brown seaweeds, as well as among different geographical areas [12]. The tannin content of our extract (54.3 mg per g) appeared similar to that showed by Dang et al., who founded a tannin content of 56.17 ± 0.22 mg per g of the Australian *Padina* sp. extract [16].

Previous studies reported the antioxidant activity of some brown algae measured with the FRAP method. Agregán et al. measured the in vitro antioxidant activity of aqueous extracts of three brown seaweed from the Atlantic Ocean, in the area of Camariñas, Spain. They found FRAP values of 7.52, 51.66 and 26.93 µmol/g of extract for *Ascophyllum nodosum*, *Fucus vesiculosus* and *Bifurcaria bifurcata,* respectively [14]. Kelman et al. quantified the antioxidant activity of methanol extracts from 37 samples of algae, comprising 30 species of Hawaiian algae from 27 different genera. They showed that the brown algae as a group had the highest mean antioxidant activity among Hawaiian algae with a mean FRAP value of 3.55 ± 3.16 μM/μg extract [15]. The antioxidant activity of our extract from French Polynesia *Padina pavonica* appeared in line with the previous works on brown algae, given that acetone is not the chosen solvent for the extraction of antioxidant compounds.

Different *Padina* species possess different sterol compositions. In French Polynesian *Padina pavonica* we tested, fucosterol appeared to be exceptionally high (7.40% of EPP) in respect of cholesterol (0.97% of EPP), leading to a ratio between fucosterol and cholesterol (F:C) of 7.6:1. Comparing our sterols pattern with literature data, we can see that F:C for *Padina pavonica* from French Polynesia is higher than what previously found in the Aegean Sea *Padina pavonica*, 0.7:1 [17], Adriatic Sea *Padina pavonica*, 0.3:1 [2] and in Turkish Mediterranean Sea *Padina pavonica* [11]. The differences found in the sterol composition could be attributed to differences in the ecological conditions, life cycle of the algae and seasonal variations. [2,18,19]. Fucosterol is the main phytosterol in brown algae [20]. Many studies have described the biological and pharmacological effects of fucosterol including antidiabetic, antioxidant, anti-inflammatory, ability to reduce blood cholesterol levels in in hyper- and normocholesterolemic subjects. Fucosterol has been demonstrated to possess pro-apoptotic activity toward several cancer cell lines (colon carcinoma [21], breast carcinoma [22], promyelocytic leukemia [21]) by promoting activation of caspase-3 [20]. These data strongly suggest the potential anti-cancer activity of EPP, analogously with what reported for *Turbinaria ornata* sterols [21] or fucosterol from *Turbinaria conoides* [23]. Furthermore, fucosterol has bone regenerative effects as demonstrated by in vivo and vitro studies [24]. In an estrogen-deficient ovariectomized (OVX) animal model, oral administration of fucosterol ameliorated several bone-quality parameters (bone mineral density, bone microarchitecture and BV/TV ratio, osteocalcin and CTx serum biomarkers [24]. At cell level, fucosterol increased MG63 osteblast-like cells proliferation, alkaline phosphatase activity and mineralization capacity, while preventing osteoclasts differentiation and RANK expression [24,25,26].

Several terpenes and terpenoid compounds were identified in *P. pavonica*. Terpenoids are considered as promising bioactive molecules in the search for anticancer drugs, due to their effect in inhibiting mitotic cell division [27]. Various diterpenes have been identified in several species of the genus *Cystoseira* with antitumoral and antioxidant activities [9]. Among these, squalene appears to influence several biochemical and physiological activities which are interesting for the treatment of cancer [28], it can suppress the growth of tumor cells, partially prevent the development of chemically-induced cancer and cause regression of some already existing tumors [29]. A rich squalene diet enhances chemoterapeutic activity by increasing immune system efficiency and by lowering blood cholesterol content [28,29]. Squalene supplementation stimulates the reticuloendothelial system, resulting in a marked increase in cellular and non-specific immune function [28]. Evidence suggests that squalene might assist in maintaining white cell counts during radiation treatment [28], and, in animal models, supplementation is associated with prolonged survival time subsequent to exposure to lethal doses of radiation [28]. Squalene’s ability to inhibit ornithine decarboxylase (ODC) is also of significant interest in cancer prevention and treatment [29]. Cancer cells are known to utilize polyamines as growth substrates, and since ODC is a rate-limiting enzyme in the generation of many of the polyamines, ongoing cancer research has been, and is currently, investigating agents with the ability to interfere with this enzyme’s activity [28].

In EPP, phytol—an acyclic monounsaturated diterpene alcohol and constituent of chlorophyll—was found in a remarkable amount. The anticancer activity of phytol against several tumor cell lines in vitro has been assessed [30,31,32], as well as its capacity to induce the apoptosis in hepatocellular carcinoma cells [33] and in human gastric adenocarcinoma AGS [34].

Other identified compounds with relevant biological activity are: neophytadiene, dihydroactinidiolide, 2,4-di-*tert*-butylphenol (DTBP) and n-Nonadecanol-1. Neophytadiene, is a terpene compound that possess a strong bactericidal, antifungal, antipyretic, analgesic, antioxidant and anti-inflammatory properties [35]; dihydroactinidiolide has cytotoxic effects against five human carcinoma cell lines and one melanoma cell line [36,37]; DTBP was found to possess potent antioxidant effect [38,39] in addition to fungicidal and cytotoxic activity against HeLa cancer cell line [40]. Finally, n-Nonadecanol-1, one of the major detected component in *Ceratonia siliqua* pods essential oil, demonstrated a strong cytotoxic effect against two cancer human cell lines, HeLa and MCF-7 [41].

As far as the FAs composition, palmitic acid appears to be the predominant FA in our extract, as in Dictyotales order brown algae [2,11,17,42,43]. Palmitic acid has been reported to have to have antioxidant, hypocholesterolmic nematicide, pesticide, antiandrogenic flavor, hemolytic and 5-alpha reductase inhibitor activity. In French Polynesian *Padina pavonica*, a significant percentage of oleic and palmitoleic acid was also detected. Oleic acid attracted attention as the Mediterranean diet, characterized by high olive oil (rich in oleic acid) consumption, has been linked to a protective effect against cancer [44]. A wide range of studies have been conducted on breast cancer, where a potential protective effect of oleic acid has been described [45,46]. In addition, epidemiological studies suggest that olive oil may have a protective effect on colorectal cancer development [47]. In this regard, animal studies have also shown that dietary olive oil prevented the development of colon carcinomas in rats, confirming that olive oil may have chemopreventive properties against colon carcinogenesis [48,49]. Oleic acid has been reported to act synergistically with cytotoxic drugs, enhancing their antitumor effect [50,51]. Eventually, palmitoleic has been demonstrated to possess antitumor effect on Ehrlich ascites tumor [52].

Although C_18_ and C_20_ PUFAs are reported to be characteristic of brown algae [2,53], the concentrations of PUFAs in our *Padina pavonica* appeared unusually low compared to PUFAs composition of Turkish Mediterranean Sea *Padina pavonica* [11], *Padina boryana* from the Saudi Arabian coast [2], or *Padina pavonica* from Jordan [53] and other characterized brown algae from the Bohai Sea [54]. By contrast, in our material, SFAs represented the prevalent percentage of total FAs (63.85% of total FAs and 41. 03% of total EPP).

The differences in EPP chemical profiles observed between present and literature data can be explained on the basis of environmental and experimental conditions. Several parameters are known to influence the composition of phenolic compounds or FAs produced by the same species of algae: stage of algal growth, harvest season, geographic location, genetic diversity etc. [11,13]. As an example, higher amount of high-quality phenolic compounds is generated during the hot climate and during the early stage of the growth in order to prevent the photooxidative damage and sea grazers [13]. In addition, also the extraction methodology (techniques, solvent, temperature, raw material) notably affect the yield of extracted compounds from a quantitative and a qualitative point of view [55].

As far as it regards the anti-OS activity of EPP, anti-OS effects of *Padina pavonica* from French Polynesia has not been clearly studied yet. Therefore, this is the first work that tests and demonstrates the anti-OS properties of *Padina pavonica* from French Polynesia.

According to our results, EPP presents good anti-proliferative and pro-apoptotic activities against two osteosarcoma cell lines, namely MNNG and SaOS-2. These two cell lines were chosen based on their intrinsic properties, as they have opposite cancer-related phenotypes. According to Lauvrak [56], MNNG cells have been defined as very aggressive in terms of tumorigenicity, colony forming ability, migration/invasion and proliferation capacity; on the contrary, SaOS-2 cells have been classified as poorly aggressive. Moreover, these two cell lines possess a different p53 mutation status, being MNNG p53 mutant and SaOS-2 p53-null [57]. This approach let us speculate on a possible mechanism of action of EPP. In the present study EPP was found to be much more active against MNNG that SaOS-2 cells suggesting that EPP may upregulate p53 expression [58] and thus induce p53-dependent apoptosis in human OS cells by the activation of extrinsic pathways. Finally, we evaluated EPP toxicity in human primary osteoblasts and found that these cells were significantly less affected by the antiproliferative and pro-apoptotic activities of EPP (data not shown)

In literature there are several works that confirm the antitumoral activities for *Padina pavonica*. The dichloromethane extract of *Padina pavonica* was found to be cytotoxic towards the KB tumor cell line. An oxysterol, (hydroperoxy-24 vinyl-24 cholesterol), was identified as being responsible for this activity [59]. Likewise, the cytotoxic and apoptotic effects of *Padina pavonica* methanol extract against human cervix (HeLa) and breast cancer (MDA-MB-453) cell lines have been reported [60]. The observed anticancer activity could be connected to the rich content of phenolics, in particular the antiproliferative effects of polyphenols were reported to be due to the regulation of apoptosis, decreased Bcl-2 levels and increased Bax, Caspase-8 and Caspase-10 levels, and Fas death receptor signalling [61]. Moreover, glycosides, sulfated polysaccharides and carotenoids of the brown seaweed were found to act as potential chemoterapeutic or chemopreventive agents through the induction of caspases or cell cycle arrest [60,62,63,64,65]. In agreement with the works above mentioned and considering the present characterization of EPP (Table 1, Table 2 and Table 3), we might assume that the main constituents responsible for the antitumoral effect—that we observed in our work—may be identified in fucosterol, in two terpenoid compounds, such as dihydroactinidiolide and phytol, of which EPP was found to be rich, and in oleic acid. Notwithstanding, it should be also considered that macroalgae extracts are complex matrices and that biological activities of such extracts might not be closely related to a specific compound but rather to the mixture of components that can act synergistically. Therefore, any biological activity can be hardly explained with the analysis of a specific algae component.

## 4. Materials and Methods

### 4.1. Collection of Padina pavonica and Preparation of the Algae Extract (EPP)

The algae (*Padina pavonica* (Linnaeus) Thivy) were collected from the coastal area of Moorea, French Polynesia, in June 2014. Fronds of mature plants (20 mm) were harvested and then rinsed with water to remove salt and the associated debris. The cleaned material was air-dried and stored in a dehumidified chamber to remove the residual moisture. Once completely dried, the algae material was ground into a fine powder and extracted with acetone in a Soxhlet extractor. After that, the mixture was filtered through filter paper and the filtrate was collected. The solution was dried by rotary evaporator and the resulting material was kept in the refrigerator until the analyses.

### 4.2. Total Phenolic Content

The quantitative assay of the total phenolic compounds was determined by the Folin-Ciocalteu method [66]. A stock standard solution of 0.5 mg/mL gallic acid was prepared in 95% methanol, and then working standard solutions were prepared in the range 0.05–0.45 mg/mL. 100 µL of standard/sample (5 mg/mL in DMSO) were mixed to 200 µL of 10% Folin-Ciocalteu reagent in DI water and 700 µL of 0.7 M Na_2_CO_3_. Each solution was prepared in triplicate. The samples and the standards were incubated for 30 min in the dark. The absorbance was red at 765 nm using a Tecan Spark 20M multimode microplate reader. The total phenolic content was expressed as mg gallic acid per gram of extract.

### 4.3. Total Flavonoid Content

The determination of the content of flavonoid compounds was carried out according to the aluminum chloride colorimetric method [67]. A stock standard solution of 1 mg/mL of quercetin was prepared in methanol, and then working standard solutions were prepared in the range of 0.05–0.30 mg/mL. 500 µL of standard/sample (1 mg/mL in DMSO) were mixed to 100 µL of 10% AlCl_3_ in 1 M potassium acetate and 3.3 mL of methanol. Each solution was prepared in triplicate. After 30 min of incubation, the absorbance was measured at 430 nm using a Tecan Spark 20M multimode microplate reader. The results were expressed as mg of quercetin per gram of extract.

### 4.4. Total Condensed Tannins Content

The determination of the content of condensed tannins was performed according to the Broadhurst vanillin-HCl method [68]. A stock standard solution of 1 mg/mL of catechin was prepared in methanol, and then working standard solutions were prepared in the range of 0.05–0.30 mg/mL. 500 µL of standard/sample (2 mg/mL in DMSO) were mixed to 3 mL of 4% vanillin solution in methanol and 1.5 mL of concentrated HCl. Each solution was prepared in triplicate. The samples and the standard were incubated for 15 min in the dark. The absorbance was red at 765 nm using a Tecan Spark 20M multimode microplate reader. The results were expressed as mg of catechin per gram of extract.

### 4.5. FRAP Scavenging Ability for the Antioxidant Activity

The determination of the antioxidant activity of the extract was performed on the method of FRAP assay [69], based on the redox reaction of the reduced oxidant Fe(III) complexed by TPTZ (2,4,6-Tris(2-pyridyl)-s-triazine). The FRAP reagent for the calibration curve was prepared mixing 0.3 M acetate buffer (pH 3.6), 0.01 M TPTZ and DI water in proportion 10:1:1, respectively. A 0.001 M FeSO_4_·7H_2_O solution in DI water was used as standard for the calibration levels. The sample was analyzed mixing 100 µL of the extract solution at 5 mg/mL in DMSO with 900 µL of DI water and 2 mL of FRAP reagent, obtained mixing 0.3 M acetate buffer, 0.01 M TPTZ and a 0.02 M FeCl_3_·6H_2_O solution in proportion 10:1:1, respectively. All the reagents were prepared fresh. Each solution was prepared in triplicate. All the solutions were incubated for 30 min in the dark. Then the absorbance was measured at 593 nm using a Tecan Spark 20M multimode microplate reader. The scavenging ability was expressed as µmol of Fe^2+^ per 100 mg of extract.

### 4.6. GC-MS Analysis

5 mg of EPP were dissolved in 50 μL of pyridine and 75 μL of bis(trimethylsilyl)trifluoroacetamide (BSTFA). The mixture was heated at 80 °C for 30 min and analysed by GC/MS and finally 3 μL were injected manually in the GC-MS.

For the analysis of the most lipophilic compounds, EPP was submitted to saponification and dispersive liquid-liquid microextraction (DLLME) [70] before the derivatization step. Briefly, 30 mg of EPP were dispersed in 2 mL of methanol; 1 mL of 2 M methanolic KOH was added and the mixture was heated in a water bath for 1 h at 80 °C shaking vigorously every 15 min. Then 1.2 mL of 1 M HCl was added and the mixture was vortex-mixed. An aliquot of 400 µL was transferred to a glass centrifuge tube and 1.6 mL of DI water was added. The DLLME was performed injecting rapidly 1 mL of a mixture of acetone (900 µL) and chloroform (100 µL) into the glass tube. The tube was closed and gently shaken by hand for 1 min. After that the tube was centrifuged at 3000 rpm for 5 min and the lower phase was collected with a microsyringe. An aliquot of 50 µL of the collected phase was mixed with 30 µL of pyridine and 30 µL of BSTFA and heated in a water bath for 30 min at 80 °C. An aliquot of 3 µL was injected manually in the GC-MS.

Analysis was performed with an Agilent 6890 Series gas chromatograph (Santa Clara, CA, USA) coupled with an Agilent 5973 quadrupole mass analyser (Santa Clara, CA, USA) equipped with the MSD ChemStation software (software version D.03.00). A Phenomenex ZB-5MS plus (30 m × 0.25 mm × 0.25 μm) column (Torrance, CA, USA) was used. The oven temperature was programmed as follow: the initial column temperature of 90 °C (1 min) was increased to 110 °C at a rate of 5 °C/min; then it was increased to 233 °C at a rate of 10 °C/min and held for 5 min; then it was increased to 300 °C at a rate of 20 °C/min and held for 5 min and then it was increased to 325 °C at a rate of 10 °C/min and held for 10 min. The total run time was 40.65 min. Helium was used as a carrier gas at a flow rate of 1.4 mL/min. The split ratio was 50:1 and the injector temperature was 285 °C. For electron ionization (EI) we used the ionization voltage 70 eV. The temperatures used were 150 °C for the MS Quad and 230°C for the MS Source. Full scan mass spectra were acquired at the mass range of 35–550 Da.

The identification of the compounds in the GC-MS chromatograms was based on a comparison of the electron ionization (EI) spectra with the NIST MS library database and, in addition, the study of the mass spectrum of each peak was carried out to further elucidate the identification of the compounds with a higher probability. Standards of fucosterol and cholesterol were also analysed to obtain the exact retention time for the identification. Considering the instrumental technique used, the discrimination between isomers was not possible in certain cases.

### 4.7. Cell Cultures

Human OS SaOS-2 (ATCC-HTB-85) and MNNG (ATCC-CRL-1547) cells were obtained from American Type Culture Collection (ATCC, Manassas, VA, USA) and cultured as described [71,72,73] in DMEM containing 10% *v/v* FBS, 100 mg/mL penicillin and 100 mg/mL streptomycin. Cultures were mainteined at 37 °C in a humidified atmosphere of 5% CO_2_. Comparative analysis was performed with cell populations at the same generation.

### 4.8. Cell Viability and Proliferation

SaOS-2 and MNNG cells were seeded in a 96-well plate at a density of 8 × 10^3^ or 3 × 10^3^ cells/well, respectively, and cultured until sub-confluence (70–75% confluence). Cells were serum starved (FBS 0.1%) for 24 h and then treated with different concentrations of EPP (3.1, 6.25; 12.5, 25, 50 100 and 200 µg/mL) in starvation medium. Controls were performed treating cells with DMSO 0.2% *v/v*, corresponding to the higher concentration of the compound. After 24 h of treatment, cells were washed with sterile PBS and MTT was added to a final concentration of 1 mg/mL. After a 3.5 h incubation, cells were lysed with 100 µL DMSO. The absorbance was measured at 550 nm and percentage of cell viability was calculated relative to control and EPP half-maximal inhibitory concentration (IC_50_) was calculated by GraphPad Prism software. The experiment was repeated three times.

Cell proliferation was evaluated by cell counting with Scepter™ 2.0 Cell Counter (Merck Millipore, Burlington, MA, USA). SaOS-2 and MNNG cells were seeded in 24-well plate at a density of 4 × 10^4^ and 3 × 10^4^ cells/well, respectively, and cultured until sub-confluence (70–75% confluence). Cells were serum starved (FCS 0.1%) for 24 h and then treated with different concentrations of EPP corresponding to IC_50_/2, IC_50_ and 2*IC_50_ in starvation medium. After 24 h of treatment, cells were washed with sterile PBS and detached by trypsin. Cells were collected in clean tubes and counted, percentage of cell proliferation was calculated relative to control.

### 4.9. Cell Morphology

Cell morphology of SaOS-2 and MNNG cells was recorded with bright field microscopy (Zeiss AxioLabA1, Oberkochen, Germany). Cell morphology images were collected after 24 h treatment with EPPat different concentrations (IC_50_/2, IC_50_ and 2*IC_50_) in starved conditions (FCS 0.1%).

### 4.10. Nuclear Staining with 4′, 6-Diamidine Phenylindole (DAPI)

SaOS-2 and MNNG cells were seeded in 8-well chambered slide at a density of 8 × 10^3^ and 6 × 10^3^ cells/well respectively. After treatment with EPP for 24 h, the slides were washed with PBS and fixed in 70% ethanol for 30 min. Finally, the slides were washed twice with PBS and mounted with fluoroshield mounting medium containing DAPI. Images were captured by fluorescence microscopy (Zeiss AxioLabA1, Oberkochen, Germany).

### 4.11. Cell Diameter Analysis

As proved by Tahara et al. [74] the Scepter 2.0 cell counter could be used to evaluate apoptosis in an accurate and reproducible way by measuring cell diameter. Cell size distributions were shown as histograms on the monitor of the Scepter™ 2.0 Cell Counter, and these data were analyzed with the Scepter™ 2.0 Software Pro computer software. Before the cell diameter were analyzed, the upper and lower gates of the counter were adjusted manually to eliminate small particles. Data regarding cell diameter are presented as the mean ± standard deviation (SD) values of triplicated experiments.

### 4.12. Annexin V/Propidium Iodide Assay

Apoptosis was detected in SaOS-2 and MNNG cells treated with EPP for 6 h, by FITC Annexin V/Dead Cell Apoptosis Kit (Molecular Probes; Invitrogen Corp., Eugene, OR, USA) following manufactures protocol. A total of 300 cells from each sample were scored by using a fluorescence microscope (Zeiss AXIO LAB AI, Oberkochen, Germany) and were assessed as viable cells (AnV−/PI−), early apoptotic cells (AnV+/PI−), late apoptosis (AnV+/PI+) and necrotic cells (AnV−/PI+).

### 4.13. Western Blot Analysis

After treatment with EPP, cells were washed with sterile PBS, lysed with RIPA buffer, added with phosphate and protease inhibitors, and then disrupted by sonication for 5 min in an ice bath. Protein concentration was assessed by BCA protein assay. 20 µg of protein were resolved by 12% SDS–PAGE and transferred onto nitrocellulose membrane. The membrane was blocked in TBS, 0.1% Tween 20, 5% *w/v* nonfat dry milk at 4 °C with gentle shaking, ON. The membrane was incubated with anti-caspase 3 (rabbit polyclonal IgG, 1:1000 Cell Signaling) and anti-GAPDH HRP-conjugated (1:50,000) primary antibodies, in the same buffer, ON at 4 °C. The blot was washed three times with PBS and incubated with anti-rabbit HRP-conjugated secondary antibody (Sigma-Aldrich, Saint Louis, MO, USA) 1:80,000 for 1 h at room temperature. The membrane was washed three times with PBS and immunoreactive bands were detected using ECL (Luminata Crescendo, Merck Millipore, Burlington, MA, USA) and images acquired by LAS4000 (GE Healthcare, Chicago, IL, USA). The optical densities of immunoreactive bands were analysed by ImageQuant TL software (GE Healthcare, Chicago, IL, USA, V 7.0) using GAPDH as a loading normalizing factor. The experiment was performed in triplicate. 

### 4.14. Statistical Analysis

Experiments were performed in triplicate. Data were expressed as mean ± SD. Differences between the values were tested for statistical analysis of variance (ANOVA) using two-tailed Student’s t-test. The values of *p* < 0.05 were considered to be statistically different to control.

## 5. Conclusions

The extract of brown algae *Padina pavonica* under investigation exhibited an interesting pharmacological potential with relevant health-protecting effects. Our findings confirmed this macroalgae as a promising and unlimited source of new functional food ingredients and bioactive compounds. Moreover, this study provides convincing and integrated evidences that EPP possesses anti-cancer properties towards osteosarcoma cell lines and can be used as a nutraceutical tool to prevent bone-related diseases or to support the current treatment protocols for osteosarcoma.

## Figures and Tables

**Table 1:** Chemical composition (%) of EPP.

	EPP (%)
	**Hydrocarbons**
Fatty acids	68.83
Squalene	0.19
Other hydrocarbons	10.86
**Total hydrocarbons**	**79.88**
	**Sterols**
Cholesterol	0.97
Fucosterol	7.40
**Total sterols**	**8.37**
	**Other compounds**
α-Tocopherol	0.17
δ-Tocopherol	0.19
Phytol	5.27
Neophytadiene	5.56
2,4-di-*tert*-butylphenol (DTBP)	0.18
2(4*H*)-Benzofuranone, 5,6,7,7a-tetrahydro-4,4,7a-trimethyl-, (*R*)-(Dihydroactinidiolide)	0.37

**Table 2:** EPP composition (%) following saponification and DLLME.

	EPP (%)
	**Hydrocarbons**
Fatty acids	17.11
Squalene	0.19
Other hydrocarbons	1.65
**Total hydrocarbons**	**18.95**
	**Sterols**
Cholesterol	4.57
Fucosterol	17.57
**Total sterols**	**22.14**
	**Other compounds**
δ-Tocopherol	0.27
Phytol	53.85
2,4-di-*tert*-butylphenol (DTBP)	2.99
6-Hydroxy-4,4,7a-trimethyl-5,6,7,7a-tetrahydrobenzofuran-2(4*H*)-one	0.99
2(4*H*)-Benzofuranone, 5,6,7,7a-tetrahydro-4,4,7a-trimethyl-, (*R*)-(Dihydroactinidiolide)	0.62
n-Nonadecanol-1	0.20

**Table 3:** Fatty acids (%) of EPP.

Fatty Acids (FAs)	EPP (%)
**Saturated fatty acids (SFAs)**	
Lauric acid 12:0	0.47
Myristic acid 14:0	0.43
Pentadecanoic acid 15:0	1.95
Palmitic acid 16:0	34.15
Stearic acid 18:0	3.25
Arachidic acid 20:0	0.74
Behenic acid 22:0	0.04
Other SFAs	2.42
**Total SFAs**	**43.45**
**Monounsaturated fatty acids (MUFAs)**	
Oleic acid (18:1 *n*-9)	7.79
Palmitelaidic acid (16:1 *n*-7 (E))	7.82
Palmitoleic acid (16:1 *n*-7 (Z))	6.29
Other MUFAs	1.76
**Total MUFAs**	**23.67**
**Polyunsaturated fatty acids (PUFAs)**	
Linoleic acid (18:2 *n*-6)	0.53
Eicosapentanoic acid (20:5 *n*-3)	0.24
Arachidonic acid (20:4 *n*-6)	0.64
Other PUFAs	0.29
**Total PUFAs**	**1.70**
**Total *n*-6 PUFAs**	**1.36**
**Total *n*-3 PUFAs**	**0.24**
**Ratio *n*-6/*n*-3**	**5.61**

**Table 4:** Apoptosis in SaOS-2 and MNNG OS cells treated with EPP at IC_50_/2, IC_50_ and 2*IC_50_ after 6 h treatment. Percentage (±SD) of non-apoptotic (AnV−/PI−), early apoptotic (AnV+/PI−) and late apoptotic (AnV+/PI+) cells are reported. Results were obtained from three different experiments in triplicate. Results were obtained from three different experiments in triplicate. *p*-values were calculated, by one-way ANOVA with post hoc Dunnett test, comparing the percentages of non-apoptotic cells (AnV−/PI−) in control and treated conditions, **** *p* < 0.0001.

**SaOS-2 OS Cells**	**Non-Apoptotic** **AnV−/PI− (%) ± SD**	**Early Apoptotis** **AnV+/PI− (%) ± SD**	**Late Apoptotis** **AnV+/PI+ (%) ± SD**	***p*-Value**
**CTR**	88.04 ± 3.33	11.17 ± 2.94	0.79 ± 0.47	
**IC_50/2_**	46.36 ± 16.31	21.80 ± 8.31	31.83 ± 8.20	****
**IC_50_**	6.11 ± 1.72	10.46 ± 1.07	83.43 ± 2.76	****
2*IC_50_	0.00	9.60 ± 3.53	90.40 ± 3.53	****
**MMNG OS Cells**	**Non-apoptotic** **AnV−/PI− (%) ± SD**	**Early Apoptotis** **AnV+/PI− (%) ± SD**	**Late Apoptotis** **AnV+/PI+ (%) ± SD**	***p*-Value**
**CTR**	93.90 ± 1.54	4.30 ± 1.06	1.78 ± 1.78	
**IC_50_/2**	25.48 ± 8.11	2.05 ± 1.26	72.46 ± 9.12	****
**IC_50_**	6.67 ± 2.89	2.99 ± 2.12	90.34 ± 4.27	****
2*IC_50_	0.69 ± 0.69	5.63 ± 5.63	93.67 ± 5.32	****
